# C.E.R.A. maintains stable hemoglobin in Latin American patients on dialysis

**DOI:** 10.1007/s11255-012-0272-3

**Published:** 2012-09-19

**Authors:** Kleyton Bastos, Luis Antonio Lucarelli, Elizabeth De Francesco-Daher, Roberto Pecoits Filho, Carlos Henríquez, Beatriz Espinoza, Ignacio Villanueva, Emma Schwedt, Ruben Schiavelli, Ricardo Correa-Rotter

**Affiliations:** 1 CLINESE-Centro de Nefrologia de Sergipe, Brazil Medserv, Sao Paulo, Brazil; 2 Hospital Universitário Walter Cantídio, Fortaleza, Brazil; 3 UFC, Fortaleza, Brazil; 4 PUC-PR, Curitiba, Brazil; 5 Centro de Diálisis de Occidente, Maracaibo, Venezuela; 6 Médica Sur Fresenius Medical Care, Mexico DF, Mexico; 7 Fundación Leonor Goelkel, Bogotá, Colombia; 8 Centro de Diálisis-Asociación Española, Montevideo, Uruguay; 9 Hospital General De Agudos Dr. Cosme Argerich, Buenos Aires, Argentina; 10 Departamento de Nefrología y Metabolismo Mineral, Instituto Nacional de Ciencias Médicas y Nutrición Salvador Zubirán, Vasco de Quiroga 15, Tlalpan, Mexico DF, 14000 Mexico

**Keywords:** Anemia, C.E.R.A., Chronic kidney disease, Hemoglobin

## Abstract

**Background:**

C.E.R.A. is a continuous erythropoietin receptor activator with characteristics that permit a once-monthly schedule of administration for the maintenance treatment for chronic kidney disease (CKD) patients. The main objective of this study was to assess the maintenance of Hb concentration with once-monthly intravenous and/or subcutaneous C.E.R.A. therapy in Latin American dialysis patients with chronic renal anemia previously treated with epoetin alfa s.c or i.v 1–3 times per week.

**Methods:**

This was a single-arm, open-label, multicenter, 32-week study of anemic patients with CKD previously treated with epoetin alfa sc or iv 1–3 times per week. After a 4-week screening period, during which mean Hb levels were maintained between 10.5 and 12.5 g/dL on their previous erythropoiesis stimulating agent, eligible patients entered a 16-week C.E.R.A. dose titration period followed by a 4-week efficacy evaluation period (EEP) and a 28-week safety follow-up. The starting dose of C.E.R.A. was based on the previous dose of epoetin alfa. Doses of C.E.R.A. were then adjusted to maintain Hb levels within ±1.0 g/dL of the reference concentration and between 10.5 and 12.5 g/dL. The Hb reference concentration was defined as the mean of all Hb levels during screening. The primary end point was the proportion of patients maintaining a mean Hb concentration (g/dL) within ±1 g/dL of their reference Hb and between 10.5 and 12.5 g/dL during the EEP.

**Results:**

A total of 163 patients from 27 centers in Argentina, Brazil, Chile, Colombia, Ecuador, Mexico, Peru, Uruguay, and Venezuela entered the treatment period and 102 completed the prescribed course of C.E.R.A. Forty-five patients (43.7 %) maintained a mean Hb concentration within ±1 g/dL of their reference Hb value and between 10.5 and 12.5 g/dL during the EEP. The median monthly dose remained constant at 120 μg during the titration period and during the EEP. On the average, there were only 2.3 dose changes per patient in 28 weeks of treatment, covering 7 C.E.R.A. scheduled administrations. 53 % of all dose changes were dose decreases, 47 % increases. A total of 10 AEs and 4 SAEs were considered to be related to the study treatment.

**Conclusions:**

Once-monthly C.E.R.A. treatment effectively maintains stable Hb concentrations in patients with chronic renal anemia undergoing dialysis with a good safety and tolerability profile.

## Introduction

Anemia is a common complication in patients with chronic kidney disease (CKD), which can adversely affect patients’ quality of life [1, 2], as well as their risk of cardiovascular events [2] and death. Particularly in Latin America, a region with limited access to health care for a high proportion of the population [3–7], and in which patients frequently enter into care at an advanced stage of disease, the effects of CKD and associated anemia have substantial impact on quality of life, morbidity, and mortality. With regard to health care access in this region, it is important to distinguish between the technological and the financial ability to provide treatment. Although the know-how and technology are present and allow adequate treatment in most of Latin America, limited funding of health providers has in conjunction with a scarce number of nephrologists in some countries, had the effect of restricting access to health care in many nations.

Erythropoiesis stimulating agents (ESAs) are effective in treating anemia and are associated with improved quality of life [1] and patient outcome [8]. However, the short dosing interval of conventional ESAs, and the more frequent injections (1–3 times per week) for epoetin alfa and epoetin beta [9], constitutes a burden for patients and puts a strain on health care resources.

One strategy to decrease the burden caused by frequent ESA administration is the development of ESAs with longer dosing intervals. C.E.R.A. is a continuous erythropoietin receptor activator with a half-life of about 130 h [10]. C.E.R.A. also has a lower binding affinity for the erythropoietin receptor and exhibits lower systemic clearance compared to conventional ESAs [10]. Together, these features permit a once-monthly schedule of administration for the maintenance treatment for CKD patients.

The hemoglobin (Hb) levels of dialysis patients vary substantially over time, even when receiving ESA maintenance treatment, and this fluctuation has been shown to be associated with increased hospitalization events and mortality [11–13]. There are a variety of causes, but the fluctuation seems to be most closely related to frequent ESA dose changes, iron treatment practices, and hospitalization/clinical complications [11, 14]. In addition, patients with advanced CKD typically present with high rates of oxidative stress, inflammation, and diminished biological capacity, which also contribute to a high rate of Hb fluctuation and associated adverse effects observed in this population [15].

In patients receiving ESA therapy, the production of erythrocytes places a high demand on functional iron supply. It is therefore important that ESA therapy be accompanied by an evaluation, and if necessary supplementation, of patient iron levels in such patients. The KDOQI clinical practice guidelines recommend assessing both the level of iron stores and the adequacy of iron for erythropoiesis by measuring serum ferritin concentration and transferrin saturation, respectively [16].

The main objective of this study was to assess the maintenance of Hb concentration with once-monthly intravenous and/or subcutaneous C.E.R.A. therapy in dialysis patients with chronic renal anemia previously treated with epoetin alfa. A secondary objective was to evaluate the safety and tolerability of intravenous and/or subcutaneous C.E.R.A.

## Patients and methods

### Patients

Patients 18 years or older who presented with chronic renal failure, an Hb concentration between 10.5 and 12.5 g/dL, and an adequate iron status (serum ferritin >100 ng/mL or *T*_SAT_ >20 % or hypochromic red cells <10 %), as well as same dose of epoetin alfa maintenance treatment for the previous 2 months were eligible for inclusion in the study. Additional eligibility criteria included regular, long-term dialysis therapy with the same mode of dialysis for at least the previous 3 months and a Kt/V of 1.8 for peritoneal dialysis or 1.2 for hemodialysis at screening. Patients were excluded from study participation if they received a blood transfusion during the previous 2 months or presented with poorly controlled hypotension, gastrointestinal bleeding, active malignant disease, hemolysis, hemoglobinopathies, folic acid deficiency, vitamin B12 deficiency, platelet count >500 × 10^9^/L or <100 × 10^9^/L, or pure red cell aplasia.

Written informed consent was provided by all subjects and an independent ethics committee approved the study. The study was conducted in accordance with good clinical practice guidelines and the Declaration of Helsinki. Subjects who wished to withdraw from the study could do so at any time without the need to justify their decision, and investigators were permitted to withdraw a subject at any time if it was felt to be in the best interest of the subject.

### Study medication

C.E.R.A. (F. Hoffmann-La Roche Ltd., Basel, Switzerland) was provided as an injectable solution in sterile prefilled syringes containing 40, 50, 60, 100, 120, 150, 200, or 250 μg each in 0.3 mL solution. C.E.R.A. was intravenously and/or subcutaneously administered once every 4 weeks (Q4W). The starting dose of C.E.R.A. for each patient was based on the dose of epoetin alfa, administered in the week preceding the switch to C.E.R.A.

Once the subjects were enrolled, patients with serum ferritin <100 ng/mL and *T*_SAT_ <20 % received iron supplementation. The choice of iron supplement and mode of administration were according to standard practice of the participating centers. Iron supplementation in individual patients was temporarily discontinued if serum ferritin increased above 800 ng/mL or *T*_SAT_ increased above 50 %.

### Study design

This was a single-arm, open-label, phase 3 study conducted at 27 centers in Latin America with the objective to investigate the efficacy, safety, and tolerability of once-monthly administration of C.E.R.A. in dialysis patients with chronic renal anemia. Participating study centers were located in Argentina, Brazil, Chile, Colombia, Ecuador, Mexico, Peru, Uruguay, and Venezuela.

**Fig. 1:**
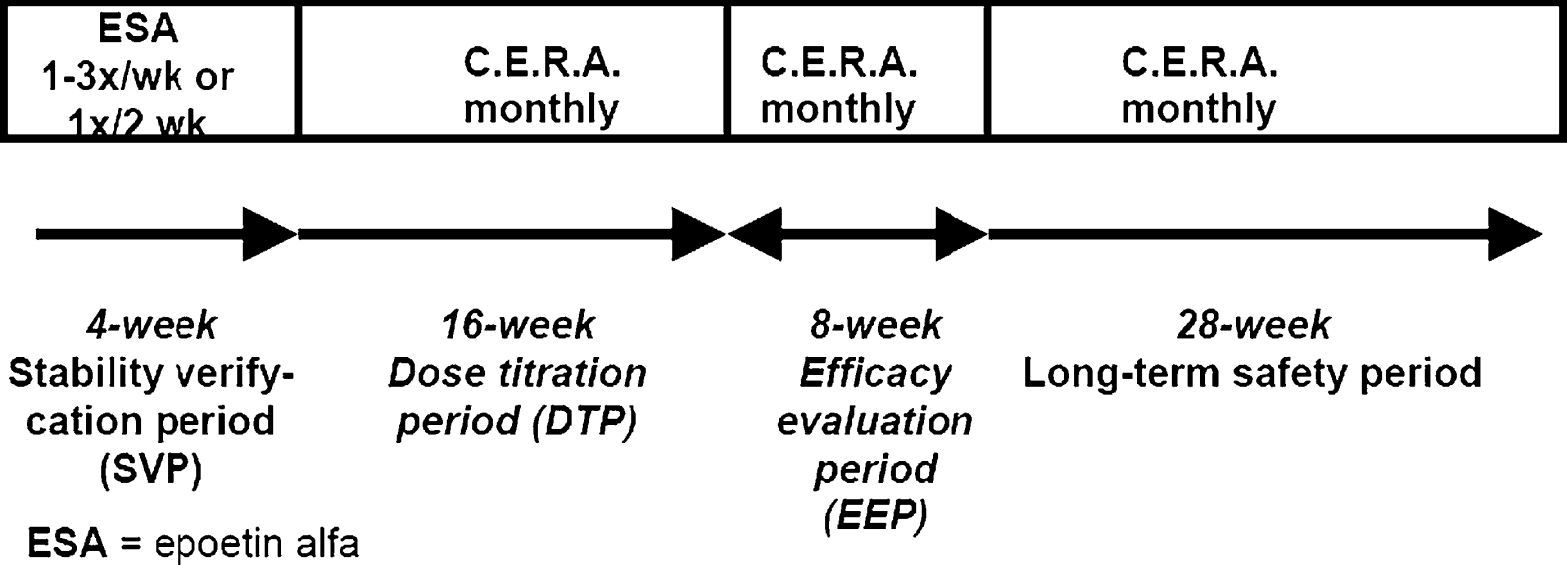
Study design

The study was divided into 4 periods (Fig. 1): a stability verification period (SVP), a dose titration period (DTP), an efficacy evaluation period (EEP), and a safety follow-up period (FU).

Following receipt of written consent, patients were screened for eligibility over a 4-week period (SVP) during which Hb was assessed weekly and patients continued to receive epoetin alfa at the same weekly dose as prior to screening. Baseline Hb was defined as the mean of all Hb values measured during the SVP and was assessed under conditions of stable epoetin alfa dosage. Patients with a stable Hb concentration between 10.5 and 12.5 g/dL were eligible to enter the DTP. Eligible patients provided a medical history including the etiology of CKD, concomitant diseases, concomitant medications, and previous treatments for anemia.

Eligible patients received a starting dose of C.E.R.A. that was based on the epoetin alfa dose administered during the week preceding the switch to the study drug. The DTP lasted 16 weeks (weeks 1–16), during which C.E.R.A. was administered every 4 weeks (i.e., weeks 4, 8, 12, and 16). Dose adjustments were made if a patient presented with a Hb concentration that was >14 or <9 g/dL, or if the difference between two consecutive Hb measurements was >2 g/dL. Dose adjustments for safety reasons were permitted at any time point during the study. An 8-week EEP (weeks 17–24) followed the DTP to assess Hb stability. A 28-week follow-up to monitor long-term patient safety followed the EEP.

### Study parameters

The primary end point was the proportion of patients maintaining a mean Hb concentration (g/dL) within ±1 g/dL of their reference Hb and between 10.5 and 12.5 g/dL during the EEP. Secondary efficacy variables included: (1) the change in mean Hb concentration between baseline and the EEP, (2) the proportion of patients maintaining an average Hb concentration within the range of 10.5–12.5 g/dL throughout the EEP, (3) the mean time spent in the Hb range of 10.5–12.5 g/dL during the EEP, and (4) the proportion of patients requiring any dose adjustment during the dose titration and evaluation periods. In addition, the overall incidence of adverse events was included in a safety evaluation.

Parameters assessed at every visit included Hb concentration, hematocrit, and vital signs. Additional assessments performed at week 4 and at the final visit (or premature withdrawal) included an electrocardiogram (ECG) and a test for anti-erythropoietin antibodies. At study weeks 8, 16, 24, 32, 40, and 48, additional laboratory, and iron parameters were assessed, including white blood cell and platelet count and the concentrations of serum creatinine, albumin, C-reactive protein (CRP), potassium phosphate, serum iron, ferritin and transferrin or total iron-binding capacity (TIBC). At week 16, patients underwent an additional physical examination and dialysis adequacy was re-assessed. For DP patients, the dialysis adequacy was evaluated weekly. Adverse events, concomitant medications, and treatments were recorded throughout the study.

### Statistical analyses

Analysis of the primary objective was performed on the per protocol (PP) population. Patients were excluded from the PP population if they had fewer than three recorded hemoglobin values during the EEP, missed an administration of C.E.R.A. in weeks 16–24, withdrew before the end of the EEP, or had inadequate iron status during the EEP, defined as a mean serum ferritin ≤100 ng/mL or a mean *T*_SAT_ ≤20 % or mean hypochromic RBCs ≥10 %. An additional analysis was performed using the data from the intent-to-treat (ITT) population to test the robustness of the results. The ITT population was comprised of patients who received at least one dose of C.E.R.A. and for whom data for at least one follow-up variable was available. Data missing at the end of the EEP was handled using the last value carried forward method (LOCF). The 95 % exact confidence interval used for the response rate was calculated using the Klopper–Pearson method. Analyses of secondary efficacy variables were performed using data from the ITT population and were only descriptive.

Statistical power estimates were based on an initial estimate of approximately 70 % from a previous study [14] and a two-sided 95 % confidence interval. In order to estimate the true percentage of patients maintaining their average Hb concentration within ±1 g/dL of their reference Hb, a sample size of 200 patients would be necessary. This would enable adverse events with a true incidence of ≥0.8 % to be detected with a power of at least 80 %.

## Results

### Study population

**Fig. 2:**
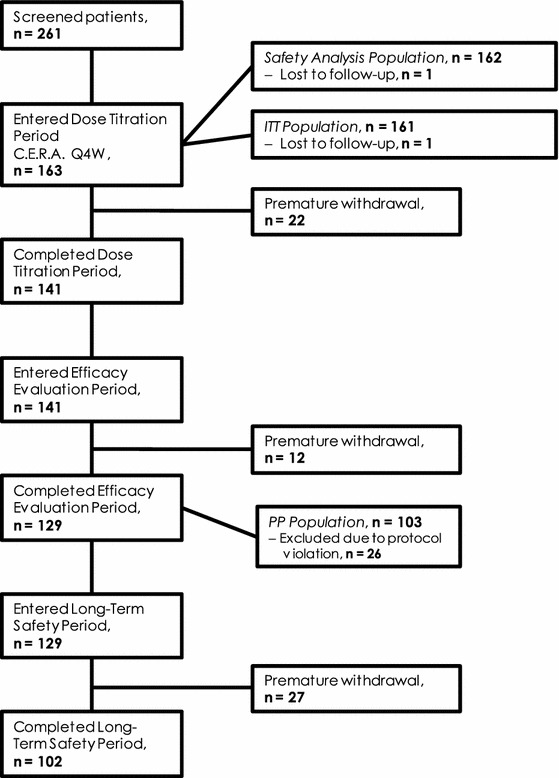
Study flow diagram and patient disposition

A total of 261 patients with CKD from 27 centers in Argentina, Brazil, Chile, Colombia, Ecuador, Mexico, Peru, Uruguay, and Venezuela were screened between October 2007 and March 2009. Of these, 163 patients entered the treatment period and 102 completed the prescribed course of C.E.R.A. medication, while 61 patients prematurely withdrew from the study (Fig. 2). The most common reasons for early withdrawal were safety-related reasons (20 patients) and protocol violations (15 patients).

The PP population comprised 103 patients and the ITT and safety populations consisted of 161 and 162 patients, respectively. The main reasons for exclusion from the PP population were having missed an administration of the study drug (*n* = 56), having fewer than 3 Hb values during the EEP (*n* = 27), requiring a blood transfusion during the DTP or EEP (*n* = 7), having a baseline Hb value not between 10.5 and 12.5 g/dL (*n* = 6), and deviation from a planned administration date by more than 10 days (*n* = 6).

The ITT population comprised 67 female (42 %) and 94 (58 %) male patients. The median age at the time of receipt of informed consent was 54 years. The most common causes of CKD in the safety population were hypertension/large vessel disease (41 %) followed by diabetic nephropathy (30 %). The mean baseline Hb concentration among patients in the safety population was 11.4 g/dL. A summary of the safety population patient characteristics at baseline, including laboratory values and iron status, is provided in Table 1.

**Table 1:** Baseline characteristics of safety population (*n* = 162)

Male, *n* (%)	94 (58)
Median age, years (range)	55 (20–92)
Median weight, kg (range)	67 (42–104)
Mean Hb, g/dL (SD)	11.4 (±0.6)
Mean serum ferritin, μg/L (SD), *n* = 157	592 (±421)
Mean iron, μmol/L (SD), *n* = 159	13.9 (±6.3)
Mean TIBC, μmol/L (SD), *n* = 97	38.5 (±10.4)
Mean transferrin, g/L(SD), *n* = 75	1.8 (±0.5)
Mean *T*_SAT_, % (SD), *n* = 135	35 (±19)
Median CRP, mg/L (min and max), *n* = 113	8.3 (0.0;816)
Median Kt/V (interquartile range), *n* = 134	1.5 (1.3–1.8)
Primary causes of CKD, *n* (%)	
Hypertension/large vessel disease	67 (41)
Diabetes	49 (30)
Undefined etiology	26 (16)
Glomerulonephritis	22 (14)
Other	17 (11)
Interstitial nephritis/pyelonephritis	6 (4)
Polycystic kidney disease	6 (4)
Other hereditary/congenital diseases	5 (3)
Neoplasms/tumors	3 (2)
Secondary glomerulonephritis/vasculitis	2 (1)

Unless otherwise indicated; *Hb* hemoglobin, *TIBC* total iron-binding capacity, *T*_*SAT*_ transferrin saturation, *CRP* C-reactive protein, *CKD* chronic kidney disease

All patients were undergoing dialysis at the time of study entry; 88 % were receiving hemodialysis and 12 % were receiving peritoneal dialysis.

Prior to the start of the DTP, all patients were receiving treatment with epoetin alfa. The median dose of epoetin alfa during the SVP was 6,000 IU per week. One hundred and eighteen patients (73 %) received iron supplements prior to study entry.

### Study treatment

The mean cumulative total dose of C.E.R.A. up to week 20 was 807 μg (±291) and the median was 720 μg. The mean monthly dose of C.E.R.A. was 135.5 μg (±40.2) during the DTP and 132.7 μg (±77.2) during the EEP. The median monthly dose remained constant at 120 μg during the titration period and during the EEP.

### Primary efficacy evaluation

**Fig. 3:**
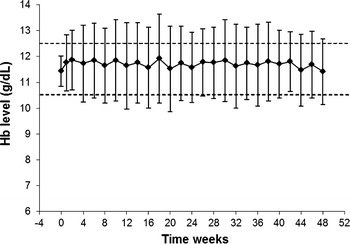
Mean Hb values (safety population)

Evaluation of the primary objective was performed on the PP population (*n* = 103). The mean Hb concentration at baseline was 11.5 g/dL (±0.57). During the EEP, the mean Hb concentration was 11.7 g/dL (±1.19) (Fig. 3). Forty-five patients (43.7 %) maintained a mean Hb concentration within ±1 g/dL of their reference Hb value and between 10.5 and 12.5 g/dL during the EEP.

### Secondary efficacy evaluations

Patient Hb concentrations in the ITT population (*n* = 161) also stayed close to baseline levels but showed slight fluctuations. The proportion of patients maintaining a mean Hb concentration within ±1 g/dL of their reference Hb value and between 10.5 and 12.5 g/dL during the EEP was 36 %. The proportion of patients who only maintained a mean Hb level within ±1 g/dL of their reference Hb value was 51 %. The mean change in the time-adjusted average of Hb concentration between baseline (SVP) and the EEP was 0.08 g/dL (±1.48). The median time spent in the Hb target range of 10.5–12.5 g/dL during the EEP was 30 days.

On the average, there were 2.3 dose changes per patient in 28 weeks of treatment, covering 7 C.E.R.A. scheduled administrations. The dose of C.E.R.A. needed to be adjusted in 106 patients (66 %) during the DTP. The dose was decreased in 39 patients, increased in 35 patients, and increased and decreased in 32 patients. Of the one hundred and forty-one patients who entered the EEP, the dose of C.E.R.A. needed to be adjusted in 72 patients (51 %), was increased in 34 patients (24 %) and decreased in 27 patients (19 %). The dose needed to be increased and decreased in 11 patients (8 %).

### Iron status

**Fig. 4:**
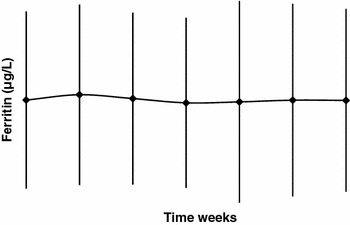
Mean serum ferritin levels (safety population)

**Fig. 5:**
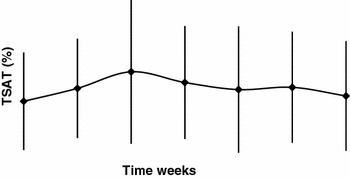
Mean transferrin saturation (*T*_SAT_) levels (safety population)

Iron supplementation was given to 58 patients (36 %) during the study. Iron supplements were primarily given in the form of saccharated iron oxide (82 % of supplemented patients), but also as iron dextran (13 %), oral iron (4 %), or ferric sodium gluconate complex (1 %). Throughout the study, the iron levels in the patients, as reflected by the mean serum ferritin concentration and the mean transferrin saturation, were maintained at a constant high level within the predetermined ranges of serum ferritin >100 and <800 ng/mL and *T*_SAT_ >20 and <50 %. High standard deviations were present, however, at all time points (Figs. 4, 5).

During the study, median CRP levels varied between 6.3 and 10.1 mg/L.

### Safety and tolerability

One hundred and twenty patients (74 %) reported 416 adverse events (AEs) that occurred during treatment with C.E.R.A. The most commonly reported AEs were hypertension (13 %), diarrhea (7 %), and hypotension (6 %). The body system classes most affected were infections and infestations (33 % of all patients); vascular disorders (25 %); gastrointestinal disorders (24 %); and injury, poisoning, and procedural complications (17 %). Nine patients experienced a total of 10 AEs that were reported by the investigators as related to the study treatment. These included one case each of anemia, constipation, diarrhea, arteriovenous fistula thrombosis, wound secretion, hemoglobin decreased, alopecia, hyperhidrosis, hypertension, and thrombosis. However, some of these may not be actually related to the study drug itself.

Seventy-eight serious AEs (SAEs) were reported in 53 patients (33 %) during the C.E.R.A. treatment period. Four SAEs were considered to be drug-related. Twenty-seven SAEs began during the titration period, 16 occurred during the EEP. Fourteen patients died after the start of C.E.R.A. treatment. All deaths were unrelated to the study drug.

Anti-erythropoietin antibodies were not detected in any patients during the study.

## Discussion

The results of this study demonstrate that stable Hb concentrations can be maintained in CKD patients on dialysis after switching to once-monthly C.E.R.A., irrespective of previous ESA treatment. In summary, 45 patients (43.7 %) maintained their mean Hb concentration within ±1 g/dL of their reference Hb value and between 10.5 and 12.5 g/dL during the EEP. In the ITT population, the proportion of patients maintaining their mean Hb concentration within ±1 g/dL of their reference Hb value and between 10.5 and 12.5 g/dL during the EEP was lower (36 %). The 2007 KDOQI anemia guidelines as well as the 2009 SLANH anemia guidelines state that the upper Hb limit during treatment should be 12 g/dL. The present protocol established 12.5 g/dL for Hb upper target, which indeed is 0.5 g/dL higher than the nowadays recommended upper limit.

The lower result for the ITT population may be attributed to the fact that more patients in the ITT population missed study drug administrations compared to the PP population.

One limitation of the study was that the route for the administration of iron supplementation was left to the discretion of the individual study centers. CKD patients on dialysis often present with high levels of inflammation and oxidative stress [17]. Consequently, the absorption of oral iron in the intestine is limited or prevented [18]. Thus, the route for the administration of iron can have a significant effect on its utilization.

The results of this study show similarities with those of other studies demonstrating that once-monthly C.E.R.A. provides stable maintenance of Hb concentrations in patients with chronic renal anemia [18–20]. Carrera et al. reported that 64.1 % of renal anemia patients undergoing hemodialysis maintained a Hb concentration ≥10.5 g/dL and within 1 g/dL of baseline values while receiving maintenance therapy with C.E.R.A [20]. This compared with only 40.1 % of patients who received darbepoetin alfa every two weeks [18]. Similarly, in a study of patients with CKD on dialysis, Sulowitz et al. found that 66.1 % of patients treated with once-monthly C.E.R.A. maintained a Hb concentration within 1 g/dL of their baseline value [21]. Levin et al. [19] found that 67 % of CKD patients on dialysis treated with epoetin maintained a Hb concentration within 1 g/dL of their baseline value over an 8-week evaluation period. In another study of CKD patients on dialysis, Spinowitz et al. [22] compared twice-monthly C.E.R.A. with 1–3 times per week epoetin alfa and found that the proportion of patients maintaining a mean Hb concentration within 1 g/dL of the baseline value was 68.5 % for C.E.R.A.-treated and 67.7 % for epoetin alfa-treated patients. Although these studies were performed in comparable patient populations, the percentage of patients who remained within 1 g/dL of their baseline value was slightly higher than in the present study (i.e., 51 % of patients).

Variability in Hb values over time is a common phenomenon in patients on dialysis and has been shown to be associated with increased mortality. Two recent studies of patients undergoing hemodialysis and stable ESA therapy in the United Kingdom and Australia found that all patients experienced at least 1 fluctuation outside target Hb levels (11–12 g/dL in Australian study and 11–12.5 g/dL in the U.K. study) and 68–73 % had at least 3 fluctuations over a 12-month period [23]. The risk associated with this fluctuation was revealed in a retrospective analysis of 35,000 hemodialysis patients: each 1 g/dL increase in Hb variability corresponded to a 33 % increase in mortality risk [13]. During the EEP of the present study, the mean fluctuation of Hb values was 0.62 g/dL. The only other study of C.E.R.A. that reported Hb fluctuation (other than an assessment of the proportion of patients whose Hb levels remained within set limits) was that of Canaud et al. They assessed Hb fluctuation by evaluating within-patient standard deviations for Hb concentration and arrived at similar results for patients treated with twice-monthly C.E.R.A. and those treated once-weekly or once every two weeks with darbepoetin alfa: 0.86 versus 0.76 g/dL during titration and 0.63 versus 0.53 g/dL during evaluation, respectively [24]. However, in the present study, fluctuation was calculated by assessing the differences between successive hemoglobin levels, and therefore, the results of the two studies cannot be directly compared.

The fact that Hb concentrations could be maintained in the desired range (in 44 % of patients) without increasing the dose of C.E.R.A. suggests that patients had adequate iron available for hematopoiesis. This is likely the result of monitoring patient iron levels and provision of iron supplements when necessary. Indeed, throughout the study, the serum ferritin and *T*_SAT_ values were adequate, however, with ample variations. A contributing factor for this variation could be the intravenously administered iron supplementation. Furthermore, one must consider that ferritin levels are influenced by inflammatory processes. Elevated CRP levels in the current study population are indicative of such inflammatory processes, which is known to affect the ferritin levels. The percentage of patients who received iron supplementation during the study was 36 %. This is substantially lower compared with reports from other studies of C.E.R.A. in CKD patients, which reported values of 92 [24], 85 [18], 82.4 [25], 77 [19], and 86 % [22]. It is also lower compared with the number of patients treated with conventional ESAs who received iron supplementation in those studies: 85 % of darbepoetin alfa-treated patients in the Carrera et al. [18] study and 93 % of darbepoetin alfa-treated patients in the Canaud et al. [24] study. The reasons for the low percentage of patients receiving iron treatment in our study compared to the other studies are not readily identifiable.

The percentage of patients who experienced at least one AE in this study (74 %) is somewhat lower than the reports (88–95 %) from other studies [18, 19, 21, 22, 24] of C.E.R.A. in similar patient populations. On the other hand, the percentage of patients experiencing SAEs was considerably lower. Seventy-eight SAEs were reported by 18 % of patients during treatment with C.E.R.A. This compares with rates ranging from 31 to 46 % reported in other studies [18, 19, 21, 22, 24]. The discrepancy is probably a consequence of the differences in study duration, patient population/severity of disease, and/or in clinic practices.

The results of this study confirm that once-monthly C.E.R.A. treatment effectively maintains stable Hb concentrations in patients with chronic renal anemia undergoing dialysis with a good safety and tolerability profile.

*LATINO Trial Investigators*: **Argentina**: Luis Gaite, Daniel Di Tullio, Pablo A. Novoa, Osvaldo Hermida, Liliana Andrade. **Chile**: Aquiles Jara, Juan Carlos Flores. **Ecuador**: Rómulo Campaña. **Mexico**: Tommaso Bochicchio, Francisco Ruiz, Alejandro Valdes. **Peru**: Edmundo Alva, Benjamín Herrada, Alberto Koga. **Venezuela**: Ana Ma Sananez. **Uruguay**: Liliana Chifflet.
